# Ecotoxicological effect of invasive S*olidago canadensis* L. and hybrid *Solidago* x *niederederi* Khek derived extracts toward selected aquatic, semi-aquatic and soil invertebrates

**DOI:** 10.1007/s11356-026-38109-9

**Published:** 2026-08-10

**Authors:** Beáta Baranová, Kristína Bednárová, Daniela Gruľová, Qaiser Javed, Mária Sabalová, Barbara Kudláčková, Lenka Svojanovská, Giuseppe Amato, Lucia Caputo, Vincenzo De Feo, Laura De Martino, Artur Pliszko

**Affiliations:** 1 Department of Ecology, Faculty of Humanities and Natural Science, University of Prešov in Prešov, 17. novembra 1, 081 16 Prešov, Slovakia; 2 Department of Fluid Phase Separations, Institute of Analytical Chemistry of the Czech Academy of Sciences, Veveří 967/97, 602 00 Brno, Czech Republic; 3 Department of Pharmacy, University of Salerno, Via Giovanni Paolo II, 132, 840 84 Fisciano, Italy; 4 Department of Plant Taxonomy, Phytogeography and Palaeobotany, Institute of Botany, Faculty of Biology, Jagiellonian University, Gronostajowa 3, 30-387 Kraków, Poland

**Keywords:** Allelochemicals, Invertebrate toxicity, Invasive *Solidago*, Asian bush mosquito, Glass worms, Earthworms, Sludge worms

## Abstract

Allelopathy is associated with biodiversity loss and assemblage changes in *Solidago* invaded plots. Although plant–allelopathic effects are well documented, little is known about the toxicity of goldenrod allelochemicals to invertebrates, despite its high biomass production and release of bioactive compounds via volatilization, root exudation or during decomposition, whereas the lack of information is especially evident for the new hybrid *S.* × *niederederi*. We conducted a laboratory experiment to assess the effect of water extracts and essential oils derived from the invasive *Solidago canadensis* and hybrid *Solidago* × *niederederi* against larvae of the blood-feeding, invasive Asian bush mosquito (*Aedes japonicus*), glass worms (larvae of non-hematophagous *Chaoborus* mosquitoes), sludge worms (*Tubifex tubifex*), and earthworms (*Eisenia fetida*), which represent aquatic, semi-aquatic and soil-dwelling invertebrates. Impact was determined on the model organism’s mortality and lethal concentration values (LC50/LC90). Except for the content of phenolic acids and saponins in the water extracts, and the representation of dominant compounds in the essential oils plus their acetylcholinesterase and butyrylcholinesterase inhibition activity were determined too. Subsequently, possible link to the toxicity was evaluated as well. Used invertebrates exhibited various sensitivities, depending on the extract type: essential oils from both goldenrod species caused high mortality of *A. japonicus* larvae. We also observed pronounced toxicity of *S. canadensis* essential oil toward glass worms. In contrast, the essential oils from invader and hybrid had no harmful effect on the earthworms or sludge worms, while both goldenrod’s water extracts caused certain mortality of *T. tubifex* and total mortality of *E. fetida*. Our analyses suggest that the toxicity of goldenrod’s allelochemicals against invertebrates may be linked to the presence of specific phenolic acids and saponins in water extracts, and the inhibitory activity of essential oils against acetylcholinesterase and butyrylcholinesterase consequently. The study thus indicates possible ecological consequences of goldenrod invasion on the impacted faunal communities. Possible usage of Solidago representative’s biomass for the bio-based pesticide development, as part of the practically focused plant invasion management, can be deduced as well.

**Graphical Abstract:**

**Figure:**
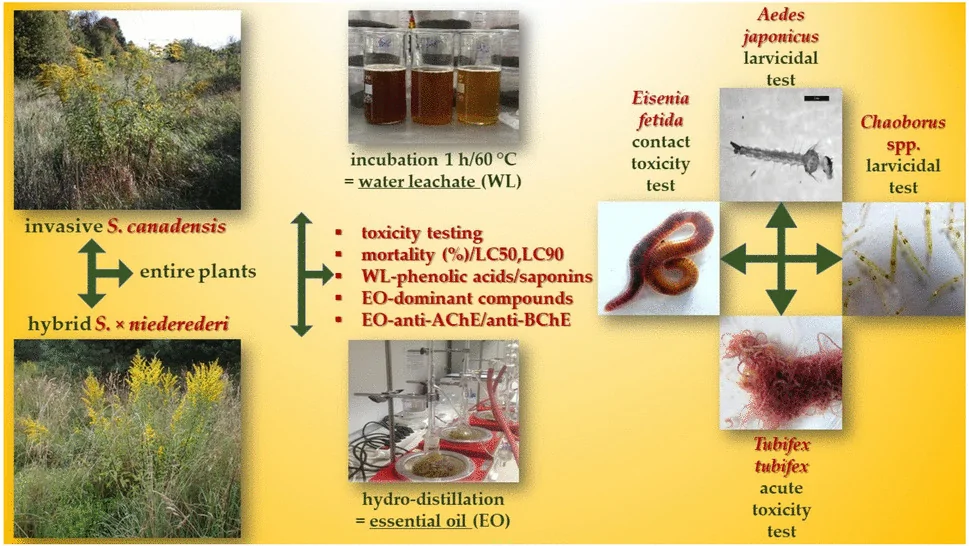

## Introduction

One of the most widespread invasive neophytes in Central and Eastern Europe is the Canadian goldenrod (*Solidago canadensis* L., Asteraceae: Astereae), a species native to North America. Its successful expansion into new areas is driven by the abundant production of small, wind-dispersed, lightweight seeds that germinate quickly and in high numbers. Its broad ecological tolerance and high resistance to pollution and environmental stress enable the species to colonize a wide range of habitats, particularly abandoned ones. For this reason, its distribution increased markedly in Slovakia after the fall of socialism in 1989. With the transition of land from state to private ownership, many plots were left without a clearly identified owner. As a result, previously cultivated areas were no longer mown or otherwise maintained. This “hands-off” management regime facilitated the development of monospecific stands of goldenrod in various sizes. (Cvachová and Gojdičová 2003; Jakobs et al. 2004; Weber and Jakobs 2005; Koutika et al. 2011; Bielecka and Królak 2019). They are commonly presented in agricultural landscapes and urban areas today and can be characterised by a gradual cyclical increase of the above and below ground goldenrod biomass.

Allelopathy is linked to biodiversity loss and the assemblage changes in the Solidago invaded plots. First and foremost, native flora—particularly annual and perennial species—are adversely affected by the invasion of goldenrods, with diversity declining by more than 60% (Bielecka et al. 2020). In addition, when *S. canadensis* comes into contact with the native European goldenrod (*Solidago virgaurea* L.), spontaneous hybridization can occur. This process has produced one of the most frequently reported alien–native hybrids in Europe, *Solidago* × *niederederi* Khek (Skokanová et al. 2020). By competing for key environmental resources such as light, water, nutrients, space, and pollinators, this hybrid threatens native plant species—including *S. virgaurea*. Moreover, it can backcross with the native species, contributing to genetic erosion and potentially leading to population decline or collapse (Lysenkov and Galkina 2023).

Shifts in native plant cover also have harmful impacts on current fauna. Changes have been observed in the communities of springtails, roundworms, ants, and beetles, leading to further negative consequences for predatory invertebrates, amphibians, reptiles, birds, and mammals (Ernst and Cappuccino 2005; Gerber et al. 2008). Goldenrod’s invasions decrease the abundance of both herbivorous ground beetles and predatory Carabidae (Kappes et al. 2007; Topp et al. 2008). However, the biomass of detritivorous groups and certain ground beetle species increases, most likely due to the substantial above- and below-ground biomass produced by goldenrods. Additionally, this biomass persists in the plots throughout the entire season, as most of it remains unharvested.

One of the factors contributing to the above-mentioned changes in plant communities is the production of allelochemicals—secondary metabolites, including essential oils. These compounds are actively released into the environment through volatilization from green plants, roots, and rhizomes during the growing phase, passively from the remained goldenrod biomass and litter during the decomposition process or via rain leachates (Song et al. 2019; Zandi et al. 2020). While the negative effects of goldenrod allelopathic substances on the germination and growth of native plants are relatively well documented (Sun et al. 2006; Abhilasha et al. 2008; Yuan et al. 2013), their impact on affected fauna has received far less attention. This lack of knowledge is especially evident for the new goldenrod hybrid. Nevertheless, considering the volume of biomass involved and the substantial production of diverse secondary metabolites, these effects should not be overlooked but carefully considered (Ferraz et al. 2022).

Secondary metabolites of invasive neophytes have been demonstrated to have the potential for utilizing as a foundation for the development of new, ecologically based biopesticides (Benelli et al. 2019a; Baranová et al. 2023, 2024). But, until the study of Martins et al. (2013) associated the essential oil of eucalyptus—released from leaves during processing—with direct toxic effects on soil invertebrates within forests converted into monospecific stands of *Eucalyptus globulus* Labill. In this study, the reproductive toxicity and feeding repellency of the essential oil against the springtail *Folsomia candida* V. Willem and the woodlouse *Porcellio dilatatus* Brandt were evaluated under laboratory conditions. The authors concluded that secondary metabolites, such as polyphenols and essential oils, are leached during leaf cuticle senescence and may accumulate in the upper soil layers. While soluble phenolic compounds and tannins tend to have relatively short persistence in soils, the effects of essential oils may be prolonged due to their hydrophobic nature and the sustained integrity of oil vesicles (Bernhard-Reversat 1993; Louzada 1997; Bernhard-Reversat et al. 2003, as cited in Martins et al. 2013).

Liu et al. (2020) conducted a controlled greenhouse experiment using *Eisenia fetida* (Savigny, 1826) to assess changes in body mass, respiration, and physiological parameters after exposure to root and leaf extracts of invasive Asteraceae species, namely *Ageratina adenophora* (Spreng.) R.M. King & H. Rob., *Bidens pilosa* L., and *Erigeron annuus* L. The study demonstrated that allelochemicals derived from these invasive plants induced reductions in body mass and respiration rates, accompanied by increased levels of oxidative stress and DNA damage biomarkers in the native earthworms.

Our own research (Baranová et al. 2023), which combined field surveys with laboratory assays, revealed a significant decline in aphid biomass in goldenrod-invaded grasslands compared with uninvaded sites. Furthermore, we confirmed the repellent activity of *S. canadensis* essential oil against aphids (*Myzus persicae* Sulzer) under laboratory conditions. Although the essential oil exhibited only moderate repellency, we propose that this volatile compound may contribute to the observed differences in aphid abundance between invaded and uninvaded grasslands, as well as potentially influencing other aboveground, plant-associated invertebrate groups.

The primary objectives of our study were to:

- assess the toxicity of water extracts and essential oils from two representatives of the *Solidago* genus—*S. canadensis* and the hybrid *S.* × *niederederi*—against four model organisms representing aquatic, semiaquatic, and terrestrial invertebrates under controlled laboratory conditions;
- compare the biological activity associated with extract type, *Solidago* taxa, and the locality of plant material origin, as well as the differential sensitivity of the tested model organisms;
- evaluate the mode of action underlying the toxicity of the extracts.

Our study can provide insight into the ecological consequences of neophyte invasions for affected faunal communities and possible usage of *Solidago* representative’s biomass for bio-based pesticide development, as part of the practically focused plant invasion management.

## Material and methods

### Plant material

Samples of *S.* *canadensis* were collected from three different localities in Slovakia: Gemerská Hôrka (GH) – south Slovakia, Hankovce (HA) – eastern Slovakia and Plaveč (PL)—northern Slovakia (Table 1), in 2022. Sampling plots included wild mixed populations with the coverage of Canadian goldenrod > 60%. The botanical identification of taxa was performed following the morphological description of Cvachová and Gojdičová (2003).

**Table 1:** List of samples—sampling plots

	GH	HA	PL
*S. canadensis*	48° 31′ 14.3978933″ N	49° 00´54.6″ N	49° 26´20.200″ N
	20° 22′ 43.3830857″ E	21° 94´53.172″ E	20° 84´47.639″ E
*S.* × *niederederi*	DK	OL	SU
	54° 05′ 007″ N	54° 01′ 136″ N	54° 06′ 126″ N
	22° 33′ 415″ E	22° 31′ 483″ E	22° 53′ 240″ E

GH-Gemerská Hôrka, HA-Hankovce, PL-Plaveč, DK-Dąbrowskie Kolonia, OL-Olecko, SU-Suwałki

Samples of *S.* × *niederederi* were collected from three different localities in north-eastern Poland: Dąbrowskie Kolonia (DK), Olecko (OL) and Suwałki (SU) (Table 1), in 2023. Sampling plots included wild mixed populations of the hybrid and its parental species, with the coverage of the hybrid < 20%. The individuals of *S.* × *niederederi* were identified based on morphological features provided by Nilsson (1976) and Gudžinskas and Žalneravičius (2016).

The localities and their corresponding results are presented as separated samples in the study.

Approximately 50 plants, including stems, leaves and inflorescences, were randomly chosen within coverage and sampled in the goldenrod full blooming period. Plants were cut into small pieces and left to freely dry in the shadow place during the 14 days. The dried material was then mixed using a kitchen mixer. Until extracts preparation, the mixtures were kept in a dry and dark place (approx. 18 °C).

### Extracts preparation

Two types of extracts were tested for their toxicity: water extracts and essential oils.

A.) Water extracts

Water extracts were prepared from 10 g of dried mixed plant material immersed in 100 mL of distilled water and incubated at 60 °C for 1 h. Extracts were filtered through a Buchner funnel with Whatman N. 1 filter paper and centrifuged at 6,000 rpm (revolutions per minute) for 30 min. Three separated water extracts were prepared from each sample (based on sampling locality). Freshly extracts were used, and, if needed, kept in the refrigerator till analysis (3–5 °C). Concentration was expressed as the content of dry biomass in mg/mL. As the water extracts were independently prepared for each experimental repetition, and, dry biomass content of individual extracts slightly differed among repetitions, the actual concentrations used for LC50 calculations were expressed as dry biomass content (mg/mL) rather than only as volumetric dilutions.

B.) Essential oils

Essential oils were extracted from eighty grams dried mixed plant material by hydro-distillation in a Clevenger-type apparatus (Kavalierglass, Sázava, Czech Rep.) at 100 °C for 3 h. The extracted oils were solubilized in *n*-hexane and stored under N_2_ at +4 °C in dark until analysis. The plant materials yielded colorless-yellow oils with a strong herbaceous, aggressive aroma. Essential oils were kept in dark glass vials in a dark cold place till analyses. The actual concentrations used for LC50 calculations were expressed in mg/mL based on the essential oils specific weight.

### Model organisms

Both extracts from *S. canadensis* and *S.* × *niederederi* were similarly tested for their toxicity, using four model organisms, representing aquatic, semiaquatic and soil invertebrates:

- 1.) Aquatic larvae of invasive Asian bush mosquito (*Aedes japonicus* Theobald, 1901, Diptera: Culicidae). Species, that originated in Japan and Korea, is recognized as one of the most widespread mosquito species globally today. Adults are biting and bloodsucking, and thus possibly transmitting several agents of serious diseases (Medlock et al. 2015). Third to fourth instar larvae were collected from the rainwater barrels in the private garden. Species was identified using Becker et al. (2010), taking into account Farajollahi and Price (2013) and Pfitzner et al. (2018). The voucher specimen is kept at the Department of Ecology, FHaNS University of Prešov in Prešov, Slovakia. Larvae were kept in the rainwater from the barrels in a refrigerator at an average temperature of 4.0 °C till experiments to eliminate their transformation into pupae.
- 2.) aquatic larvae of *Chaoborus* spp. genus (Diptera: Chaoboridae)—non-biting mosquitoes, etc. “*glass worms*” representing harmless water organisms. Larvae were commercially obtained in the pet store.
- 3.) semiaquatic worm *Tubifex tubifex* Müller, 1774 (Annelida, Oligochaeta: Tubificidae)—tubificid segmented worm (sludge worm, savage worm) representing harmless water organisms. The species is often used as standard model organism in ecotoxicological studies. Worms were commercially obtained in the pet store.
- 4.) soil earthworms *Eisenia fetida* Savigny, 1826 (Annelida, Oligochaeta: Lumbricidae)—redworm representing harmless ground organisms. Earthworms came from our own breeding – adult specimens at least five centimeters long were used for the bioassay.

### Extracts toxicity assesment

Three volumetric dilutions of water extracts were used in all toxicity tests for every model organism: undiluted, 100% extracts, and extracts diluted with 60% and 30% (v/v) (see Extracts preparation). For the dilution, distilled water was used, and applied as control as well. Four doses of essential oils were used in all tests for every model organism: 0.04, 0.07, 0.1, and 0.13 µl/mL (see Extracts preparation). For the dilution, 2% aqueous dimethylsulfoxide (DMSO, 99.9% purity, Ing. Petr Švec – PENTA, Praha, Czech Republic) was used, and applied as control as well.

Final concentrations were selected based on a preliminary fit-range test for *Ae. japonicus* to cover the full dose–response spectrum, representing low, medium, and high effect levels corresponding approximately to 0–20% mortality, around 50% mortality (close to LC50), and 80–100% mortality, respectively.

For each tested dilution (water extracts: 30%, 60%, and 100%; essential oils: 0.04, 0.07, 0.10, and 0.13 µL/mL), three technical replicates, each containing 10 individuals, were performed within a single experimental run (*n* = 90 for water extracts; *n* = 120 for essential oils). The entire experiment was independently repeated three times under identical laboratory conditions, resulting in a total sample size of *n* = 270 individuals for water extracts and *n* = 360 individuals for essential oils for every model organisms.

### 1.) test of toxicity to *Aedes japonicus *and* Chaoborus* spp.

To evaluate water extract/essential oil toxicity, i.e. larvicidal activity against Asian bush mosquito and *Chaoborus* spp. larvae were tested using the standard methodology suggested by the World Health Organization (WHO 2025), with slight modification following Brito et al. (2021). Organisms were exposed to the three dilutions of water extracts (30%, 60%, and 100%) and four doses of essential oils (0.04, 0.07, 0.10, and 0.13 µL/mL) (see above). Experiments were incubated at 25 ± 2 °C. Larvae mortality was assessed after 24 h. Larvae were considered dead when did not spontaneously move, even if subjected to mechanical stimulation with a gentle brush.

### 2.) test of toxicity to *Tubifex tubifex*

To evaluate water extract/essential oil toxicity against *T. tubifex*, sludge worms were subjected to an express 3-min acute toxicity test following Tichý et al. (2007). Organisms were exposed to the three dilutions of water extracts (30%, 60%, and 100%) and four doses of essential oils (0.04, 0.07, 0.10, and 0.13 µL/mL) (see above). If no negative response was observed after the first 3 min, the exposition was prolonged. Then, the number of dead worms was also checked after 10, 20, 30, 60, 180, 240 min and after 24 h. Experiments were incubated at 25 ± 2 °C.

### 3.) test of toxicity to *Eisenia fetida*

To evaluate water extract/essential oil toxicity against *E. fetida*, redworms were subjected to contact toxicity test according to OECD 1984. Organisms were exposed to the three dilutions of water extracts (30%, 60%, and 100%) and four doses of essential oils (0.04, 0.07, 0.10, and 0.13 µL/mL) (see above). One individual was placed in a Petri dish equipped with filter paper soaked with 2 mL of relevant water extract/essential oil dilution. Mortality was evaluated after 30, 60, 90 min and 48 h consequently. Experiments were incubated at 25 ± 2 °C.

### Water extracts parameters and saponins content determination

In aim to explain the mode of water extracts toxicity action, the content of phenolic acids (namely Caffeic acid, Ferulic acid, Chlorogenic acid, P-coumaric acid, P-hydroxybenzoic acid, Protocatechuic acid and Syringic acid) representation and saponins content were determined.

The extracts were separated using an Agilent 1200 series HPLC–DAD system (Agilent Technologies, USA) equipped with a Zorbax SB-C18 column (4.6 mm × 150 mm, 5 µm particle size; Agilent Technologies, USA) maintained at 30 °C throughout the run. The mobile phase consisted of acetonitrile (A) and 0.1% formic acid (B), following a gradient program: 0 min: 5% A; 15 min: 60% A; 20 min: 100% A; 20–30 min: 100% A. Prior to each run, the column was equilibrated with a mobile phase containing 5% A for 10 min. The flow rate was set at 0.7 mL/min, and an injection volume was 5 µL. Detection of analytes was carried out at 260, 280, and 360 nm. The target compounds were identified by comparing their retention times with those of authentic standards and the standard addition method. The internal standard method was used for quantitative analysis.

Calibration levels were prepared by diluting a concentrated mixture of individual phenolics (1 mg/mL) by pipetting 2.5–100 µL of concentrated analytes and filling up to 1 mL with methanol. A 40 µl aliquot of internal standard solution (5 mg/mL) was added to each calibration level and each of these solutions was injected in triplicate into HPLC–DAD system. The calibration curves were constructed by linear regression of the peak-area ratio of the individual standard to the internal standard versus the concentration. Good linearity with a correlation coefficient of *r*^*2*^ ˃ 0.998 for all compounds was achieved in the concentration range studied. The limit of detection (LOD, *s/n* = *3*) and limit of quantification (LOQ, *s/n* = *10*) were in the range of 0.040–0.100 µg/mL and 0.133–0.333 µg/mL, respectively. The repeatability of the method, characterized by a relative standard deviation (RSD, (%)), was RSD < 1% for all analyzed compounds.

To express saponins content in the goldenrods plant material, the shake foam test was performed (Mietlińska et al. 2019). 10 ml of boiling water was added to 0.5 g of plant powder in a calibrated cylinder, and the mixture was then left to cool. The cylinder was then violently shaken for 10 s. The height of the produced foam was measured immediately at the moment of generation. The procedure was repeated three times for each sample; the mean of the foam layer’s height in millimeters was used.

### Essential oils parameters and cholinesterases inhibition determination

To assess mode of essential oils toxicity action, composition of essential oils and acetylcholinesterase and butyril cholinesterase inhibition were determined.

GC/MS analyses were conducted on an Agilent 6850 Ser. II apparatus (Agilent, Santa Clara, CA, USA), equipped with a DB 5 fused silica capillary column (30 m × 0.25 mm i.d., 0.33 µm film thickness), connected to an Agilent Mass Selective Detector MSD 5973; the ionization energy was set at 70 eV and the electron multiplier voltage energy was 2000 V. Mass spectra (MS) were acquired over a mass range of 40 to 500 amu with a scan rate of 5 scans/s. The column temperature was programmed as follows: 40 °C, with 5 min initial hold, and then to 270 °C at 2 °C/min, 270 °C (20 min); injection means, without split (1 L of a 1:1000 *n*-hexane solution); injector and detector temperatures were fixed to 250 °C and 290 °C, respectively. Annotation of essential oil components was performed by comparing their mass spectra with those reported in the literature (Adams, 2007; Davies, 1990; Johnston, 1989; Linstrom and Mallard, 2001) and confirmed by comparing their experimentally determined Kovats retention indices (KI). In order to calculate the relative retention indices (KI), a homologous series of *n*-alkanes (C_10_–C_35_) was used as a reference. Further confirmation was achieved by comparing their mass spectra with those of authentic compounds available in our laboratory and injected under the same condition. The components’ relative concentrations were obtained by peak area normalization. Response factors were not considered.

In the study, we present and for the data processing we use only the predominant essential oils compounds with representation >5%.

Both used mosquito species are representatives of the insect class (*Insecta*). Some insecticides exert their toxicity acting on the enzyme acetylcholinesterase (AChE) and/or butyril cholinesterase (BChE) reducing their activity (Mladenović et al. 2018). Therefore, acetylcholinesterase and butyril cholinesterase inhibition (anti-AChE and anti-BChE) activity indicate insecticidal potentialities (Kobenan et al. 2022) and could explain, in part, the mode of action of used essential oils. The determination of essential oil anti-AChE and anti-BChE activity was subsequently provided in this relationship. Cholinesterases inhibition was studied by Ellman’s method (Ellman et al. 1961) with following modifications. Four hundred 15 μL of Trizma-HCl buffer 0.1 M (pH 8), 10 μL of different concentrations of EOs dissolved in DMSO (99.9% purity, Ing. Petr Švec – PENTA, Praha, Czech Republic) solution 1%, and 25 μL of 0.28 U/mL of AChE or BchE (purchased by Sigma Aldrich) solution were placed for 15 min at 37 °C. Then, 75 μL of a 1.83 mM solution of AChI or BChI and 475 μL of DTNB was added. After 30 min at 37 °C the mix was transferred in 1 mL cuvette and the samples were read at 405 nm in a spectrophotometer (Thermo Fischer Scientific, Vantaa, Finland). Galantamine was the reference drug.

### Data processing

The vast majority of studies evaluate plant extract activity focusing on the larvicidal (mosquitos) or nematocidal (worms) potential, where extract activity is expressed through mortality and lethal concentrations (LC50/LC90). Therefore, to express the toxicity of extracts we used, we applied a similar procedure.

*S. canadensis* and *S.* × *niederederi* toxicity was assessed on the basis of:

- 1.) model organism mortality (in %) and its distinction from control,
- 2.) lethal concentrations (LC50/LC90).

To compare between water extract and essential oil toxicity and between separated samples, based on the plant material origin, LC50/LC90 were calculated for each experimental replicate. Then, to express general toxicity for every sample (locality of plant material origin) and to compare between *S. canadensis* and *S. *× *niedrederi*, data from all three replicates were pooled together to calculate average mortality and LC50/LC90 values. LC50 and LC90 values were determined on the basis of mortality data subjected to Finney’s probit analysis with 95% confidence intervals of upper/lower confidence limit (UCL/LCL), R^2^, Chi-square and degree of freedom (Mekapogu 2021). The mutual comparison was provided using a one-way analysis of variance (ANOVA) followed by Tukey’s pairwise test performed in PAST 4.03c. (Hammer et; al. 2001). A level of significance *p* ≤ 0.05 was taken as significant.

Pearson correlation was used to assess relationship between model organisms mortality (obtained with the highest concentrations of water extracts/essential oils) and lethal concentrations (LC50), and the content of phenolic acids and saponins (for water extracts)/representation of essential oils dominant compounds and IC50 (AChE)/IC50 (BChE) (for essential oils).

Model organisms mortality (obtained with the highest concentrations of water extracts), lethal concentrations (LC50), content of phenolic acids and saponins were subjected to Hierarchical Cluster Analysis (HCA), Ward’s method and Principal Components Analysis (PCA) to understand the similarity/distance of toxicity between *S. canadensis* and *S.* × *niederederi* based on the water extracts.

Model organisms mortality (obtained with the highest concentrations of essential oils), lethal concentrations (LC50), representation of dominant compounds and IC50 (AChE)/IC50 (BChE) were subjected to Hierarchical Cluster Analysis (HCA), Ward’s method and Principal Components Analysis (PCA) to understand the similarity/distance of toxicity between *S. canadensis* and *S.* × *niederederi* based on the essential oil. Upper named original variables were normalized and standardized prior to analyse (Farouk 2024) and subjected to Hierarchical Cluster Analysis (HCA), Ward’s method and Principal Component Analysis (PCA). As there was zero or 100% mortality of *T. tubifex* and *E. fetida* after applying water extracts or essential oil, and LC50 was not determined, they were not included in the analyses. All analyses were performed in PAST 4.03c (Hammer et al. 2001).

## Results and discussion

### Toxicity to *Aedes japonicus*

The undiluted, 100% water extracts from *S. canadensis* exhibited significant larvicidal activity against *Ae. japonicus*, resulting in average mortality ranging from 93.33% to 100% for separated samples. Larvae mortality with 30% and 60% (v/v) extracts dilutions was also high, ranging between 43.33–68.89% and 72.22–95.53% consequently. Compared to the control, larvae mortality was always significantly (*p* < 0.05) higher. LC50 values ranged from 4.12 to 5.88 mg/mL, LC90 from 9.86 to 17.51 mg/mL consequently (Table 2).

**Table 2:** Larvicidal activity of two types of extracts from invasive *S. canadensis*, collected from three localities in Slovakia, against invasive mosquito *Ae. japonicus*

*S. canadensis*		LC50 LCL–UCL (95% confidence limit)	LC90 LCL–UCL (95% confidence limit)	R^2^	Chi-test (χ2) Sig	df
*Ae. japonicus* larvae						
Water extracts	GH	4.12 (3.21–5.29) mg/mL	9.86 (7.67–12.66) mg/mL	0.847	0.991	4
	HA	4.21 (3.41–5.21) mg/mL	10.38 (8.39-−12.85) mg/mL	0.695	0.57	5
	PL	5.88 (4.60–7.51) mg/mL	17.51 (13.71–22.37) mg/mL	0.815	0.991	6
Essential oil	GH	0.037 (0.028–0.048) mg/mL	0.069 (0.053–0.089) mg/mL	0.988	0.787	1
	HA	0.031 (0.022–0.044) mg/mL	0.072 (0.051–0.101) mg/mL	0.979	0.977	2
	PL	0.042 (0.034–0.052) mg/mL	0.067 (0.054–0.083) mg/mL	0.998	0.994	1

*GH* Gemerská Hôrka, *HA* Hankovce, PL-Plaveč

After exposure to the highest *S. canadensis* essential oils dilutions, the mortality of *Ae. japonicus* was significant as well, and ranged from 91.11% to 97.78%. The lowest dilutions caused larvae mortality > 35% on average. Compared to the control, larvae mortality was always significantly (*p* < 0.05) higher. LC50 values ranged between 0.031 and 0.042 mg/mL, LC90 between 0.067 and 0.072 mg/mL consequently (Table 2).

Larvicidal activity of undiluted, 100% water extracts from hybrid *S.* × *niederederi* was lower, resulting in mortality ranging from 31.11% to 46.67%. Lower dilutions caused mortality ranging between 14.45–16.67% (30% dilution) and 16.67–28.87% (60% dilution). Mortality was, in every case, significantly (*p* < 0.05) higher in comparison to 2% DMSO as control. LC50 values ranged from 35.21 to 51.17 mg/mL, LC90 from 172.39 to 463.87 mg/mL consequently (Table 3).

**Table 3:** Larvicidal activity of two types of extracts from hybrid *S.* × *niederederi*, collected from three localities in north-eastern Poland, against invasive mosquito *Ae. japonicus*

*S.* × *niederederi*		LC50 LCL–UCL (95% confidence limit)	LC90 LCL–UCL (95% confidence limit)	R^2	Chi-test (χ2) Sig	df
*Ae. japonicus* larvae						
Water extracts	DK	35.21 (22.69–54.64) mg/mL	252.20 (162.50–391.42) mg/mL	0.806	0.968	7
	OL	51.17 (31.08–84.24) mg/mL	463.87 (281.74–763.74) mg/mL	0.832	0.977	7
	SU	28.27 (18.86–42.36) mg/mL	172.39 (115.03–258.36) mg/mL	0.612	0.737	7
Essential oil	DK	0.053 (0.040–0.069) mg/mL	0.113 (0.086–0.147) mg/mL	0.879	0.077	2
	OL	0.039 (0.032–0.049) mg/mL	0.071 (0.058–0.087) mg/mL	0.971	0.523	2
	SU	0.043 (0.034–0.053) mg/mL	0.077 (0.062–0.096) mg/mL	0.945	0.798	2

The highest dilutions of *S.* × *niederederi* essential oil used caused the following mortality of Asian bush mosquito larvae: 65.55%, 80.00% and 81.11%. The lowest dilutions caused only low larvae mortality, ranging between 2.22 and 13.33%. Then, mortality was significantly higher in comparison to 2% DMSO as control with an exception concerning the lowest essential oil dilutions. LC50 values were as follows: 0.053, 0.039 and 0.043 mg/mL, LC90 0.071, 0.077 and 0.113 mg/mL consequently (Table 3).

DK-Dąbrowskie Kolonia, OL-Olecko, SU-Suwałki

The vast majority of studies evaluate plant extract activity focusing on the larvicidal (mosquitos) or nematocidal (worms) potential. For this reason, and to express the toxicity of extracts used by us, we predominantly compare our results with these studies.

One of the very first and innumerable studies using Asian bush mosquito (*Ae. japonicus*) evaluated larvae mortality after the exposition to various concentrations of *Syzygium aromaticum* L. (Myrtaceae) essential oil (Reuss et al. 2020). The results indicate approximately doubled larvicidal activity of clove essential oil in comparison to those from *Solidago* we used, as the LC50 and LC90 were as follows: 0.017 mg/mL and 0.047 mg/mL respectively. 100% larvae mortality was observed by 0.080 mg/mL.

The mosquito larvicidal activity of the essential oil from *S. canadensis* leaves was previously reported by Benelli et al. (2019a) as well. However, the study used *Culex quinquefasciatus* (Say, 1823) as the target mosquito species. Nevertheless, the maximal tested essential oil concentration, i.e. 100 μl L^−1^ caused 61.0% mortality of southern house mosquito larvae, resulting in LC50 of 89.3 μl/L and LC90 of 189.6 μl/L. The values (after conversion into millimeters and taking 1 µl as 1 mg approximately—values were recalculated for comparative purposes using approximate equivalence between µL and mg, acknowledging differences in oil density. Converted values were used only for comparison purposes) indicate stronger mosquitocidal activity of *S. canadensis* and *S.* × *niederederi* essential oils used in our study. Distinctions can, at least, result from possibly higher sensitivity of *Ae. japonicus* larvae to goldenrod essential oil*.* Furthermore, we used whole plants including inflorescences, since they are known to produce higher essential oil yield and with higher insecticidal activity in comparison to the leaves (Baranová et al. 2023). Different geographical origins of plant material can also play a role.

Compared to our previous study with Asian bush mosquito (Baranová et al. 2024), *S. canadensis* essential oil shows a bit stronger larvicidal activity than essential oil from *Heracleum mantegazzianum* (Sommier & Levier) seeds, as LC50 and LC90 were 0.067 and 0.179 mg/mL consequently. However, water extracts from *S. canadensis* were doubled stronger in comparison to those from Giant hogweed seeds, with LC50 9.235 mg/mL and LC90 32.88 mg/mL. On the other side water extracts and essential oils from *S.* × *niederederi* showed expressively lower larvicidal activity than *H. mantegazzianum* according to LC50 values. Raghavendra et al. (2013) and Prathibha et al. (2014) used *S. canadensis* leaves extracts against *Aedes aegypti* L., as another of the Aedes genus representant. The obtained LC50 and LC90 values were expressively lower than ours. However, petroleum ether was used for extraction, which leads to the high content of biologically active compounds in comparison to water extracts.

Essential oil from aerial parts of two subspecies of *Tanacetum argenteum*, as other representatives of Asterales: Asteraceae (same as *Solidago*), i.e. *T. argenteum* (Lam.) Willd. subsp. argenteum (Lam.) and *T. argenteum* (Lam.) Willd. subsp. *canum* (C. Koch) Grierson were applied in the larvicidal bioassays using *Ae. aegypti* (Ali et al. 2014). *T. argenteum* subsp. *argenteum* showed LC50 value of 0.093 mg/mL, whereas *T. argenteum* subsp. *canum* killed only 40% of the larvae at the highest dose of 0.125 mg/mL. When compared, essential oil from Canadian goldenrod as well as the hybrid showed stronger larvicidal activity in our study. The same paid for the larvicidal activity of our *Solidago* water extracts, when comparing with the results of Cheah et al. (2013): their study tested larvicidal activity of hexane extract from *Artemisia annua* L., representatives of Asterales: Asteraceae as well, and so against *Ae. aegypti*, *Anopheles sinensis* Wiedemann, 1828 and *Cx. quinquefasciatus*. The extract showed just moderate larvicidal effects against mosquitoes, with the LC50 values 0.25, 0.28, and 0.38 mg/mL for *An. sinensis*, *Ae. aegypti*, and *Cx. quinquefasciatus* respectively. *An. sinensis* was found to be the most susceptible species, whereas *Cx. quinquefasciatus* was the most tolerant to the *A. annua* extract.

Comparing our results to larvicidal activity of Temephos—an organophosphate larvicide used to treat water infested with disease-carrying insects, obtained in the studies of Vivekanandhan et al. (2020) and Sugauara et al. (2022), larvicidal activity of *S. canadensis* and *S.* × *niederederi* essential oils would be very similar against *Anopheles stephensi* Liston 1901 (LC50 = 0.030 mg/mL), however weaker if using *Cx. quinquefasciatus* (LC50 = 0.007 mg/mL for both) (Vivekanandhan et al. 2020), but stronger when using *Ae. aegypti* L. according to Sugauara et al. (2022) (LC50 = 40 mg/mL).

### Toxicity to* Chaoborus* spp.

Undiluted, 100% water extracts from *S. canadensis* caused lower mortality of *Chaoborus* spp. larvae when compared to *Ae. japonicus* in general. Glass worms mortality ranged between 21.67 and 28.34% for separated samples. Lower dilutions caused mortality ranging between 10.00–13.34% (30% dilutions) and 15.00–18.33% (60% dilutions), whereas mortality was significantly higher than control only by 60% and 100% water extracts. LC50 values were 23.80, 65.1 and 215.3 mg/mL respectively (Table 4). For GH leachate, mortality response against glassworms remained relatively low and irregular across the tested concentration range. Consequently, the probit model showed poor fit and the LC90 estimate should be interpreted cautiously, while for comparative purposes, LC50 value remains more informative.

**Table 4:** Larvicidal activity of water extracts from invasive *S. canadensis*, collected from three localities in Slovakia, against non-biting mosquito of the *Chaoborus* genus

*S. canadensis*		LC50 LCL–UCL (95% confidence limit)	LC90 LCL–UCL (95% confidence limit)	R^2	Chi-test (χ2) Sig	df
*Chaoborus* larvae						
Water extracts	GH	215.3 (51.7–896.5) mg/mL	28 615.7 (6 873.5–11 9132.8) mg/mL*	0.131	0.058	3
	HA	23.80 (16.30–34.75) mg/mL	81.42 (55.76–118.89) mg/mL	0.862	0.393	3
	PL	65.1 (34.8–122.0) mg mg/mL	792.8 (423.2–1 485.00) mg/mL	0.526	0.329	4

*GH* Gemerská Hôrka, *HA* Hankovce, PL-Plaveč

*LC90 estimated outside the experimentally observed mortality range

In the opposite*, S. canadensis* essential oil showed extreme toxicity to glass worms: regardless of essential oil dose/sample (locality) 100% mortality was observed, even when the set of tests was completely repeated. Thus, no *Chaoborus* LC50 was noted for *S. canadensis* essential oils.

Undiluted, 100% water extracts from hybrid *S.* × *niederederi* caused following *Chaoborus* spp. larvae mortality: 17.78%, 22.22% and 27.78% for separated samples.

With 30% and 60% dilutions, mortality ranged between 10.00–12.23% and 13.33–24.44% consequently. Mortality was, in every case, significantly higher in comparison to 2% DMSO as control. LC50 values ranged from 51.17 to 35.92 mg/mL (Table 5).

**Table 5:** Larvicidal activity of water extracts and essential oils from hybrid *S.* × *niederederi*, collected from three localities in north-eastern Poland, against non-biting mosquito of the *Chaoborus* genus

*S.* × *niederederi*		LC50 LCL–UCL (95% confidence limit)	LC90 LCL–UCL (95% confidence limit)	R^2	Chi-test (χ2) Sig	df
*Chaoborus* larvae						
Water extracts	DK	35.92 (23.11–55.85) mg/mL	258.22 (166.09–401.44) mg/mL	0.794	0.958	7
	OL	51.17 (31.08–84.24) mg/mL	463.87 (281.74–763.74) mg/mL	0.832	0.977	7
	SU	40.21 (27.87–58.02) mg/mL	172.32 (119.44–248.61) mg/mL	0.911	0.892	6
Essential oil	DK	0.082 (0.049–0.139) mg/mL	0.252 (0.149–0.427) mg/mL	0.99	0.927	2
	OL	0.042 (0.032–0.055) mg/mL	0.082 (0.063–0.107) mg/mL	0.988	0.847	2
	SU	0.042 (0.034–0.051) mg/mL	0.069 (0.056–0.085) mg/mL	0.957	0.596	2

*DK* Dąbrowskie Kolonia, *OL* Olecko, *SU* Suwałki

The highest *S.* × *niederederi* essential oil dilutions caused the following mortality of *Chaoborus* larvae: 65.56%, 91.11%, and 92.22% for separated samples. The lowest dilutions caused only low larvae mortality, ranging between 3.33 and 16.67%. Then, mortality was significantly higher in comparison to 2% DMSO as control with an exception concerning the lowest essential oil dilutions. LC50 values ranged between 0.042 and 0.082 consequently mg/mL (Table 5).

Water extracts from both, the invader, and hybrid as well, had relatively low adverse effects on glass worms, in contrast to the stronger toxicity observed for *Heracleum mantegazzianum* seed water extracts in our previous study (Baranová et al. 2024). Similarly, Jiang et al. (2018) reported that *Chaoborus crystallinus* De Geer, 1776 exposed to saponin extracts from Quillaja, tea, and quinoa did not reach 50% mortality even at 0.2 mg/mL (LC50 > 0.2 mg/mL). By comparison, essential oils, particularly from *S. canadensis*, exhibited markedly stronger negative effects. This pattern is consistent with observations in other Asteraceae species; for instance, *Matricaria chamomilla* L. essential oil caused lower toxicity to *Chaoborus plumicornis* (Fabricius, 1794), with an LC50 of 0.502 mg/mL (Satyal et al. 2015), supporting the generally higher biological activity of essential oils compared with water-based extracts across plant taxa.

### Toxicity to *Tubifex tubifex*

Among the two extract types, only water extracts exhibited measurable activity against sludge worms. No toxic effects were observed for essential oils, regardless of *Solidago* species or the applied dilutions. Even prolonged exposure up to 24 h did not result in any mortality of sludge worms.

After applying *S. canadensis* 100% undiluted water extracts, mortality of sludge worms ranged from 20.00 to 24.44% for separated samples, 30% and 60% dilutions caused mortality ranging between 0 to 1.11% and 1.11 to 7.78% consequently. Mortality differed significantly in comparison to control only by the undiluted, 100% water extracts. LC50 values ranged from 23.03 to 52.19 mg/mL consequently (Table 6).

**Table 6:** Anthelmintic activity of water extracts from invasive *S. canadensis*, collected from three localities in Slovakia, and hybrid *S.* × *niederederi*, collected from three localities in north-eastern Poland, against sludge worms

Water extracts		LC50 LCL–UCL (95% confidence limit)	LC90 LCL–UCL (95% confidence limit)	R^2	Chi-test (χ2) Sig	df
*T. tubifex*						
*S. canadensis*	GH	48.63 (32.33–73.16) mg/mL	211.87 (140.84–318.74) mg/mL	0.505	0.085	5
	HA	23.03 (18.31–29.04) mg/mL	46.26 (36.73–58.27) mg/mL	0.826	0.653	4
	PL	52.19 (32.46–83.92) mg/mL	236.52 (147.11–380.29) mg/mL	0.49	0.025	4
*S.* × *niederederi*	DK	13.31 (11.74–15.08) mg/mL	21.94 (19.36–24.87) mg/mL	0.901	0.69	7
	OL	12.63 (11.08–14.40) mg/mL	21.46 (18.83–24.46) mg/mL	0.856	0.54	5
	SU	20.25 (16.59–24.28) mg/mL	44.51 (37.12–53.36) mg/mL	0.855	0.156	5

*GH* Gemerská Hôrka, *HA* Hankovce, *PL* Plaveč, *DK* Dąbrowskie Kolonia, *OL* Olecko, *SU* Suwałki

*S.* × *niederederi* 100%, undiluted water extracts caused sludge worm mortality ranging between 71.11 and 97.78% of separated samples; 30% and 60% dilutions caused mortality ranging between 0 to 4.44% and 1.11 to 50% consequently. However, exposition prolonged to 24 h led to 100% mortality by all water extracts dilutions used. Mortality differed significantly in comparison to control only by the undiluted 100% and 60% dilutions of water extracts. LC50 values ranged between 12.63 and 20.25 mg/mL consequently (Table 6).

### Toxicity to *Eisenia fetida*

In strong contrast, *E. fetida* exhibited the greatest sensitivity to the water extracts among all tested model organisms. Exposure to extracts from both *Solidago* species resulted in 100% mortality, irrespective of the dilution applied. These findings were fully reproducible, as the repeated test series yielded identical outcomes. Moreover, mortality of the majority of exposed individuals occurred within approximately 30 min, despite the assay duration being set at 48 h.

The strong toxicity of water extracts toward *E. fetida* is likely attributable to saponins, consistent with previous reports, identifying *S. canadensis* and *S. gigantea* as saponin-rich species (Mietlińska et al. 2019). This aligns with Kamal et al. (2017), who observed an average mortality time of 29.65 min for *Pheretima posthuma* Vaillant, 1869 exposed to crude saponin extracts from *Atriplex laciniata* L.. Saponins are known for their potent anthelmintic activity and toxicity to aquatic organisms (Jiang et al. 2019; Kamal et al. 2017). Concentrations as low as 0.002–0.003 mg/mL induce 50% mortality in *T. tubifex* and *Lumbricus variegatus* Müller, 1774 (Jiang et al. 2018), confirming earthworms as highly sensitive taxa.

Augustin et al. (2012, in Jiang et al. 2018) explain saponins’ ultimate toxicity as follows: “detergent-like structures, cause damages to cell membranes by creating pores, vesiculation and membrane domain disruption.” That is why so-called soft skin organisms, just like earthworms and sludge worms, are the most sensitive to saponins.

In our laboratory bioassay, earthworms were exposed directly to pure plant extracts with the known contents of dry biomass, phenolic acids, saponins. However, the concrete amount of toxic compounds—released into the environment under natural conditions—actively and during decomposition—remains unknown. Phenolic compounds are reported to degrade rapidly in soil, while essential oils may persist due to their hydrophobic nature and the stability of oil vesicles (Bernhard-Reversat 1993; Louzada 1997; Bernhard-Reversat et al. 2003, cited in Martins et al. 2013). Given the high biomass production and litter retention of goldenrods throughout the growing season, the potential subsequent ecotoxicity should not be undoubtedly overlooked (Song et al. 2019; Zandi et al. 2020). We suppose these effects are likely to be spatially patchy, depending on invader density and organic matter accumulation, and temporally variable, influenced by leaching, precipitation, snowmelt, temperature, and decomposition dynamics.

Our suggestions can be supported by our own, yet unpublished data from a 14-day artificial soil test in which *E. fetida* was exposed to 7-day extracts of Canadian goldenrod, while those findings are consistent with Liu et al. (2020). They reported reductions in body mass and respiration, along with increased oxidative stress and DNA damage biomarkers in *E. fetida* following exposure to 48-h water extracts of leaves and roots of invasive Asterids (*Ageratina adenophora*, *Bidens pilosa*, and *Erigeron annuus*) under controlled greenhouse conditions. Notably, these effects were observed at extracts dry matter concentrations of 6.25–12.5 mg/mL, whereas the extracts used in our actual study contained an average of ≥15 mg/mL dry biomass.

In contrast, no mortality of *E. fetida* was observed following exposure to essential oils, regardless of *Solidago* species or dilution. This aligns with Benelli et al. (2019a), who reported that essential oils from the leaves of *S. canadensis* and *S. gigantea* did not induce mortality in adult earthworms. Similarly, essential oil from *Schizogyne sericea* (L. f.) DC. (Asteraceae) was non-toxic to *E. fetida* adults when tested as a non-target organism alongside four insect pests (Benelli et al. 2019b). Even prolonged exposure in 14-day artificial soil tests did not result in significant mortality (Vasantha-Srinivasan et al. 2018; Pavela et al. 2020).

By contrast, hydrolate from *Artemisia absinthium* L. (Asteraceae) produced markedly different results, causing high mortality and strongly affecting the survival of *E. fetida* during a 14-day artificial soil exposure (Pino-Otín et al. 2019).

### Mutual comparisons

Essential oils exhibited significantly stronger larvicidal activity against *Aedes japonicus* and *Chaoborus* spp. compared with water extracts (*p* < 0.05), while having no effect on earthworms or sludge worms. In contrast, water extracts caused high mortality in *E. fetida* and moderate mortality in *T. tubifex* for both invasive and hybrid *Solidago* species, consistent with our previous observations for giant hogweed (Baranová et al. 2024).

Despite the relatively high geographical distance between sampling plots, no significant differences in lethal concentrations were observed among localities for either extract type. Comparisons between species revealed that water extracts of the invasive *S. canadensis* were significantly more effective against *Ae. japonicus* (*p *< 0.01), whereas no goldenrod species differences were observed for *Chaoborus* spp.. However, essential oil from the invasive species was more potent against *Chaoborus* spp.. The hybrid *S.* × *niederederi* extracts showed significantly (*p* < 0.05) higher activity against *T. tubifex* compared with the invasive one, while no differences were detected in earthworm assays.

Overall, these results indicate that extract type, species identity, and target organism strongly influence the observed bioactivity.

### Water extracts—phenolic acids representation, saponins, corelations, PCA and HCA analyses

Representation of the seven phenolic acids in the water extracts from Canadian goldenrod and hybrid are listed in Table 7.

**Table 7:** Average representation (± SD) of phenolic acids (µg/g) and saponins (mm) in the water extracts from invasive *S. canadensis*, collected from three localities in Slovakia, and hybrid *S.* × *niederederi*, collected from three localities in north-eastern Poland

		*S. canadensis*			*S.* × *niederederi*		
		GH	HA	PL	DK	OL	SU
Caffeic acid	µg/g	1 294.9 ± 225.9	985.8 ± 42.3	732.2 ± 21.1	194.3 ± 5.6	192.6 ± 0.8	296.5 ± 3.2
Ferulic acid	µg/g	1 598.7 ± 161.3	750.3 ± 9.7	3 82.1 ± 130.1	2 723.8 ± 76.4	3 197.6 ± 117.8	3 977.9 ± 135.7
Chlorogenic acid	µg/g	3 181.4 ± 210.4	1 405.9 ± 1.9	5 587.7 ± 294.6	5 699.2 ± 265.2	7 097.6 ± 217.8	6 955.2 ± 235.9
P-coumaric acid	µg/g	163.5 ± 0.5	112.1 ± 3.2	258.8 ± 12.1	221.2 ± 4.3	222.0 ± 13.1	245.2 ± 11.7
P-hydroxybenzoic acid	µg/g	48.7 ± 2.2	52.8 ± 1.2	47.6 ± 1.8	23.8 ± 0.0	29.4 ± 0.7	21.7 ± 0.9
Protocatechuic acid	µg/g	109.9 ± 8.0	102.5 ± 2.1	84.28 ± 2.4	0	0	0
Syringic acid	µg/g	0	0	0	69.5 ± 0.9	41.5 ± 1.0	91.4 ± 4.0
Saponins	mm	54.33 ± 5.13	52.33 ± 2.52	75.0 ± 5.0	16.0 ± 1.73	31.0 ± 3.61	30.0 ± 8.67

*GH* Gemerská Hôrka, *HA *Hankovce, *PL* Plaveč, *DK* Dąbrowskie Kolonia, *OL* Olecko, *SU* Suwałki

The water extracts were dominated with Chlorogenic, followed by Ferulic, and Caffeic acid, being the most abundant compounds across all samples. *S. canadensis* showed particularly higher levels of Caffeic acid, whereas *S.* × *niederederi* generally exhibited higher Chlorogenic and Ferulic acid contents. Protocatechuic acid was present in *S. canadensis* but absent in all *S.* × *niederederi* samples at all, while Syringic acid was detected only in the hybrid. Saponin content varied markedly among samples with the highest values observed in GH.

We observed following significant correlations: Caffeic acid content was positivelly correlated with mortality of *Aedes japonicus* larvae, but negatively with mortality of *T. tubifex*, whereas P-hydroxybenzoic acid showed the very opposite trend. Saponins content was positivelly correlated with larvicidal effect (LC50) against *Ae. japonicus*, but with no association to *Chaoborus* spp.

We take into account, that the present study does not demonstrate a direct causal relationship between Caffeic acid content, P-hydroxybenzoic acid, saponins and larvicidal activity.

The heightened sensitivity of *Aedes* larvae is likely due to saponin-induced reduction of water surface tension, which interferes with siphon-mediated respiration and leads to suffocation, while the lower susceptibility of glass worms reflects their different way breathing.

Fourteen original variables (Table 8) were analyzed by PCA and were reduced to two principal components, representing 85.17% of the total variance. In particular, the first component (PC1) explains 68.46% of the total variability, the second (PC2) explains 16.71% of the total variance.

**Table 8:** Loadings of the significant variables on two first principal components from data analysis

Variables	PC1	PC2
Caffeic acid	0.29644	0.07615
Ferulic acid	−0.20999	0.32156
Chlorogenic acid	−0.25967	0.22580
P-coumaric acid	−0.19257	0.37899
P-hydroxybenzoic acid	−0.28214	−0.32652
Protocatechuic acid	0.36584	0.08099
Syringic acid	−0.28579	−0.00545
Saponins	0.21540	0.24073
Aj50	0.36668	0.04193
Cha50	0.18385	−0.41959
Tt50	0.11481	−0.52427
Aj-m	0.35668	0.16179
Cha-m	−0.03762	0.06788
Tt-m	−0.33455	−0.20387
Eigenvalue	1.80	0.44
Total variance (%)	68.46	16.71

Abbreviations and notes: Aj50/Cha50/Tt50 = LC50 of water extracts for *Ae. japonicus/Chaoborus* spp. larvae/*T. tubifex*; AJ-m/Cha-m/TT-m = mortality of *Ae. japonicus/Chaoborus* spp. larvae and *T. tubifex* after application of the 100%, undiluted water extracts

PC1 (68.46% of total variance) showed positive loadings for caffeic acid, protocatechuic acid, saponins, LC50 values for *Ae. japonicus* larvae, *Chaoborus* spp. larvae, and *Tubifex tubifex*, as well as mortality of *Ae. japonicus*, indicating their positive association with this component. In contrast, negative loadings were observed for mortality of *Chaoborus* spp. larvae and *T. tubifex*, indicating an opposite pattern of variation along PC1. PC2 (16.71% of total variance) was mainly characterised by positive loadings of most variables, whereas P-hydroxybenzoic acid, syringic acid, LC50 values for *Chaoborus* spp. larvae and *T. tubifex*, and sludge worms mortality showed negative associations, contributing to separation along this axis.

**Fig. 1:**
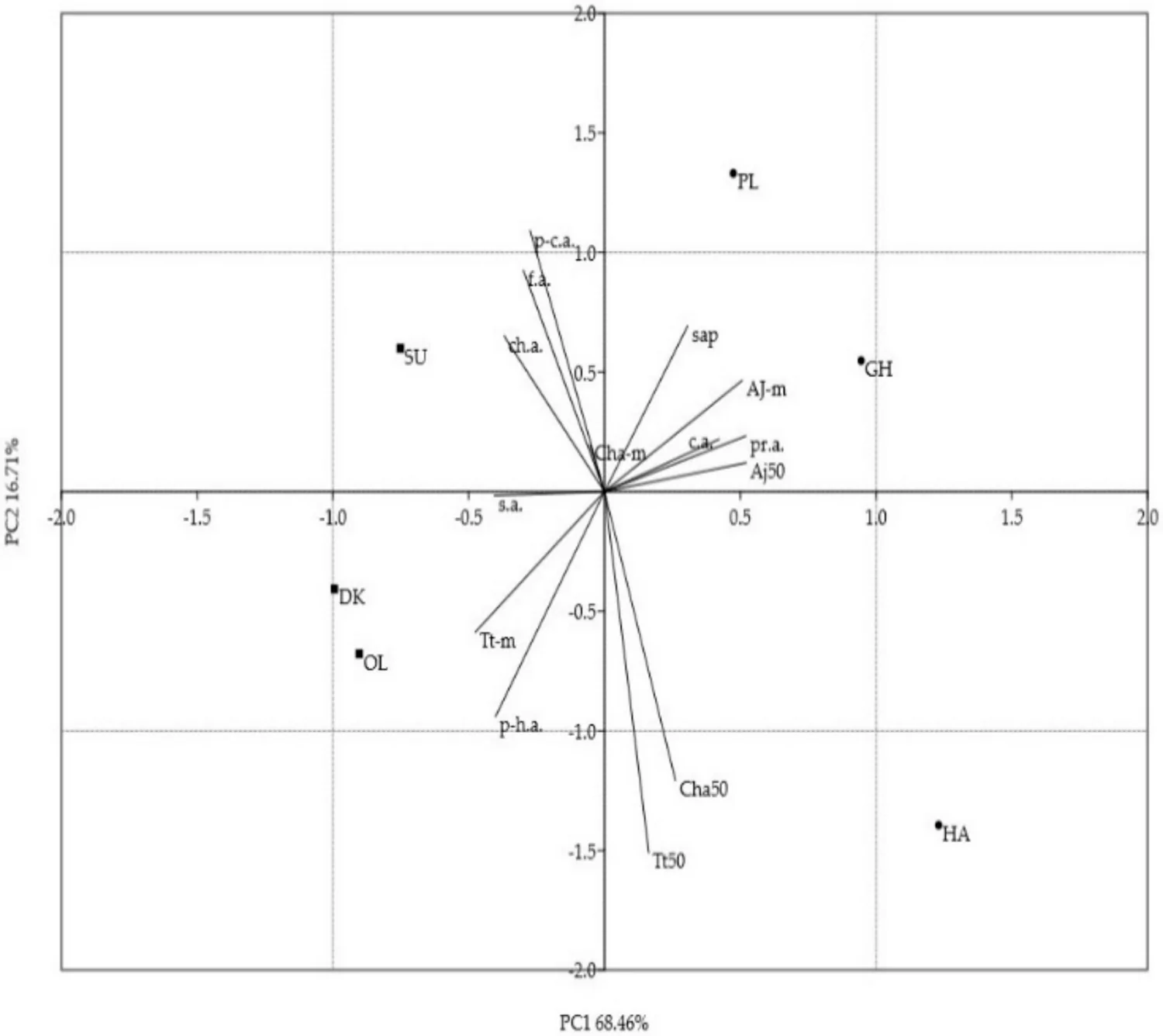
Principal Component Analysis (PCA) biplot showing the distribution of *S. canadensis* (GH, HA, PL) and *S.* × *niederederi* (DK, OL, SU) samples based on their chemical composition—representation of seven phenolic acids and saponins in water extracts and associated response variables. Abbreviations and notes: GH, Gemerská Hôrka; HA, Hankovce; PL, Plaveč; DK, Dąbrowskie Kolonia; OL, Olecko; SU, Suwałki; Aj50/Cha50/Tt50 = LC50 of water extracts for *Ae. japonicus*/*Chaoborus spp*./*T. tubifex*; Aj-m/Cha-m/Tt-m = mortality of *Ae. japonicus*/*Chaoborus spp.*/*T. tubifex* after application of the 100%, undiluted water extracts; c.a., Caffeic acid; f.a., Ferulic acid; Ch.a, Chlorogenic acid; p-c.a., P-coumaric; p-h.a, P-hydroxybenzoic acid; pr.a., Protocatechuic acid; s.a., Syringic acid; sap., saponins

**Fig. 2:**
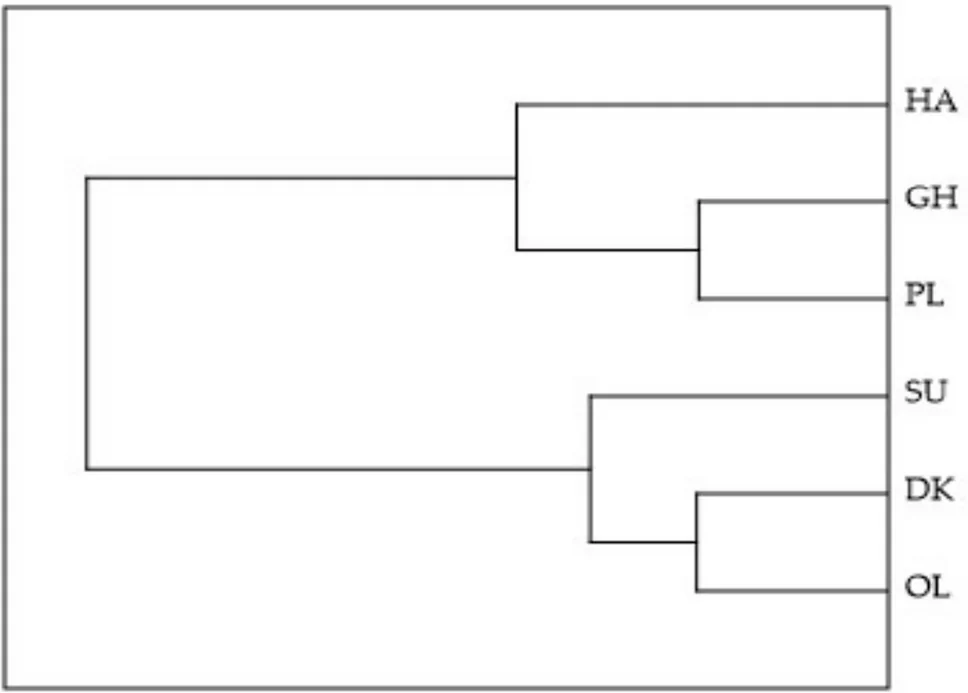
Dendrogram obtained by Hierarchical cluster analysis, Ward’s method of *S. canadensis* (GH, HA, PL) and *S.* × *niederederi* (DK, OL, SU) samples based on their chemical composition—representation of seven phenolic acids and saponins in water extracts. Abbreviations and notes: GH, Gemerská Hôrka; HA, Hankovce; PL, Plaveč; DK, Dąbrowskie Kolonia; OL, Olecko; SU, Suwałki

Principal component analysis (PCA) further indicated that *S. canadensis* localities were mainly associated with the positive side of PC1, while hybrid samples (*S.* × *niederederi*) were positioned on the negative side (Fig. 1). Hierarchical Cluster analysis, Ward’s method supported this pattern by revealing two distinct groups corresponding to *S. canadensis* and *S.* × *niederederi* localities (Fig. 2).

The performed analyses indicated a clear separation between *S. canadensis* and *S.* × *niederederi*, suggesting that differences in the compounds representation may contribute to the observed variation in water extracts activity.

### Essential oils—dominant components representation, cholinesterases inhibition, corelations, PCA and HCA analyses

Dominant compounds (representation > 5%) of essential oils from Canadian goldenrod and its hybrid are listed in Table 9.

**Table 9:** Representation (%) of dominant compounds in the essential oils from invasive *S. canadensis*, collected from three localities in Slovakia, and hybrid *S.* × *niederederi*, collected from three localities in north-eastern Poland

RT	Compound	GH	HA	PL	DK	OL	SU	KI^a^	KI^b^
60 238	15-Hydroxy-α-muurolene	5.01	-	-	5.41	-	-	1647	1775
40 278	Bornyl acetate	8.24	10.74	14.73	5.76	-	8.14	1314	1275
57 041	Caryophyllene oxide	-	-	-	-	17.54	8.56	1589	1578
66 359	Cyperenone	16.38	8.69	5.78	-	-	-	1761	1717
62 047	Germacra-4(15)0.5.10(14)-trien-1β-ol	-	-	-	8.64	-	6.20	1680	1686
50 747	Germacrene D	-	-	7.19	12.05	-	15.3	1481	1480
58 974	Isospathulenol	-	-	-	-	5.90	-	1624	1623
57 685	Ledol	-	-	-	-	11.43	-	1600	1599
21 346	Limonene	-	5.52	-	-	-	-	1044	1064
56 459	Spathulenol	7.04	-	-	18.52	-	5.56	1579	1571
28 619	*trans*-Verbenol	-	-	5.70	-	12.93	5.46	1145	1172
13 095	α-Pinene	-	6.40	-	-	-	7.86	931	941
63 125	β-Turmerone	8.89	7.30	-	-	-	-	1699	1701
53.100	γ-Cadinene	-	8.48	-	-	-	-	1521	1506

*GH* Gemerská Hôrka, *HA* Hankovce, *PL* Plaveč, *DK* Dąbrowskie Kolonia, *OL* Olecko, *SU* Suwałki, *RT* retention time, *KI*^*a*^Kovats retention index determined on a DB-5 column; *KI*^*b*^ Kovats retention index from the literature

The most abundant compounds include sesquiterpenes such as Bornyl acetate, Caryophyllene oxide, Germacrene D, and Spathulenol, with their distribution varying considerably among the samples. Some compounds (e.g., Limonene, Ledol, and γ-cadinene) were detected only in specific samples, indicating substantial chemical variability among the analyzed essential oils.

The IC₅₀ values (Table 10) for the two enzymes studied, highlighted differences both between the species and with respect to the two cholinesterases tested: *S. canadensis* samples showed generally significantly (*p* < 0.01) higher activity of acetylcholinesterase and butyrylcholinesterase in essential oil in comparison to those from the hybrid. In particular, for *S. canadensis* EOs, all samples displayed a good inhibitory activity against AChE, with IC₅₀ values ranging from 5 to 10.8 mg/mL. PL was the most active sample (IC₅₀ = 5 ± 0.20 mg/mL), probably for a higher content of bioactive compounds responsible for AChE inhibition. A similarly activity was observed against BChE, with IC₅₀ values ranging from 3.23 to 6.33 mg/mL, also in this case the most active was PL sample.

**Table 10:** Acetylcholinesterase and butyrylcholinesterase activity in the essential oil samples from *S. canadensis* and *S.* × *niederederi*

		IC50 (AChE)	IC50 (BChE)
*S. canadensis*	GH	10.8 ± 1	6.33 ± 0.15
	HA	7 ± 0.53	3.63 ± 0.12
	PL	5 ± 0.20	3.23 ± 0.21
*S.* × *niederederi*	DK	55.7 ± 2.43	20.63 ± 0.75
	OL	39.5 ± 0.79	34.9 ± 2.02
	SU	36.7 ± 1.87	21.39 ± 0.75

*GH*Gemerská Hôrka, *HA* Hankovce, *PL* Plaveč, *DK* Dąbrowskie Kolonia, *OL* Olecko, *SU* Suwałki

*S.* × *niederederi* EOs exhibited weaker activity respect *S. canadensis* EOs. The IC50 values for AChE ranging from 36.7 to 55.7 mg/mL and a similar trend was observed for BChE, with IC50 values ranging from 20.63 to 34.9 mg/mL. SU sample showed slightly stronger inhibitory activity toward both AChE and BChE compared to the other two populations.

This data could explain the possible mode of action of *S. canadensis EOs* against invasive mosquito *Ae. japonicus.* Probably, *S.* × *niederederi* EOs acted in different way against *Ae. japonicus* and non-biting mosquito of the *Chaoborus* genus*.*

No correlations were detected between IC₅₀ values for AChE or BChE and LC50/mortality of *Aedes japonicus* or *Chaoborus* spp. However, significant negative correlations (*p* < 0.05) were observed between IC₅₀ (AChE/BChE) and the relative content of Bornyl acetate in the essential oils, what can indicate, that higher concentrations of this compound corresponded to lower IC₅₀ values and, consequently, greater inhibitory activity.

This pattern coincided with the generally stronger larvicidal activity observed for *S. canadensis* essential oil, which also tended to contain higher amounts of Bornyl acetate than *S.* × *niederederi*. Nevertheless, these findings should be interpreted with caution, as the present study does not demonstrate a direct causal relationship between Bornyl acetate content, cholinesterase inhibition, and larvicidal activity.

Sixteen original variables (Table 11) were analyzed by PCA and were reduced to two principal components, representing 70.65% of the total variance. In particular, the first component (PC1) explains 40.69% of the total variability, the second (PC2) explains 29.96% of the total variance.

**Table 11:** Loadings of the significant variables on two first principal components from data analysis

Variables	PC1	PC2
15-Hydroxy-α-muurolene	0.09643	−0.34525
Bornyl acetate	−0.17244	−0.15915
Caryophyllene oxide	0.16218	0.35753
Cyperenone	−0.2844	−0.10361
Germacra-4(15)0.5.10(14)-trien-1β-ol	0.29445	−0.25805
Germacrene D	0.24843	−0.21207
Isospathulenol	0.11561	0.36370
Ledol	0.11561	0.36370
Limonene	−0.23232	−0.00842
Spathulenol	0.19971	−0.29358
*trans*-Verbenol	0.12625	0.36209
α-Pinene	−0.09374	−0.01950
β-Turmerone	−0.32818	−0.10904
γ-Cadinene	−0.23232	−0.00842
IC50 (BChE)	0.27811	0.20953
IC50 (AChE)	0.33942	−0.02141
Aj50	0.24895	−0.14724
AJ-m	−0.25946	0.12680
Cha-m	−0.27663	0.17022
Eigenvalue	1.33	0.98
Total variance (%)	40.69	29.96

Abbreviations and notes: Aj50 = LC50 of essential oil for *Ae. japonicus*; AJ-m/Cha-m = mortality of *Ae. japonicus/Chaoborus* spp. after application of the highest essential oil dose; IC50 (BChE) = butyrylcholinesterase inhibition; IC50 (AChE) = acetylcholinesterase inhibition

PC1 (40.69% of total variance) showed positive loadings for 15-Hydroxy-α-muurolene, Caryophyllene oxide, Germacra-4(15)0.5.10(14)-trien-1β-ol, Germacrene D, Isospathulenol, Ledol, Spathulenol, trans-Verbenol, IC50 (BChE), IC50 (AChE), and LC50 for *Ae. japonicus* larvae, indicating their positive association with this component. In contrast, negative loadings on PC1 were observed for Bornyl acetate, Cyperenone, Limonene, α-Pinene, β-Turmerone, γ-Cadinene, as well as for mortality of *Ae. japonicus* and *Chaoborus* spp. larvae, indicating an opposite pattern of variation. PC2 (29.96% of total variance) was mainly characterized by negative loadings of most variables, while Caryophyllene oxide, Isospathulenol, Ledol, trans-Verbenol, and mortality of both tested invertebrates showed positive associations, suggesting their contribution to differentiation along this axis.

**Fig. 3:**
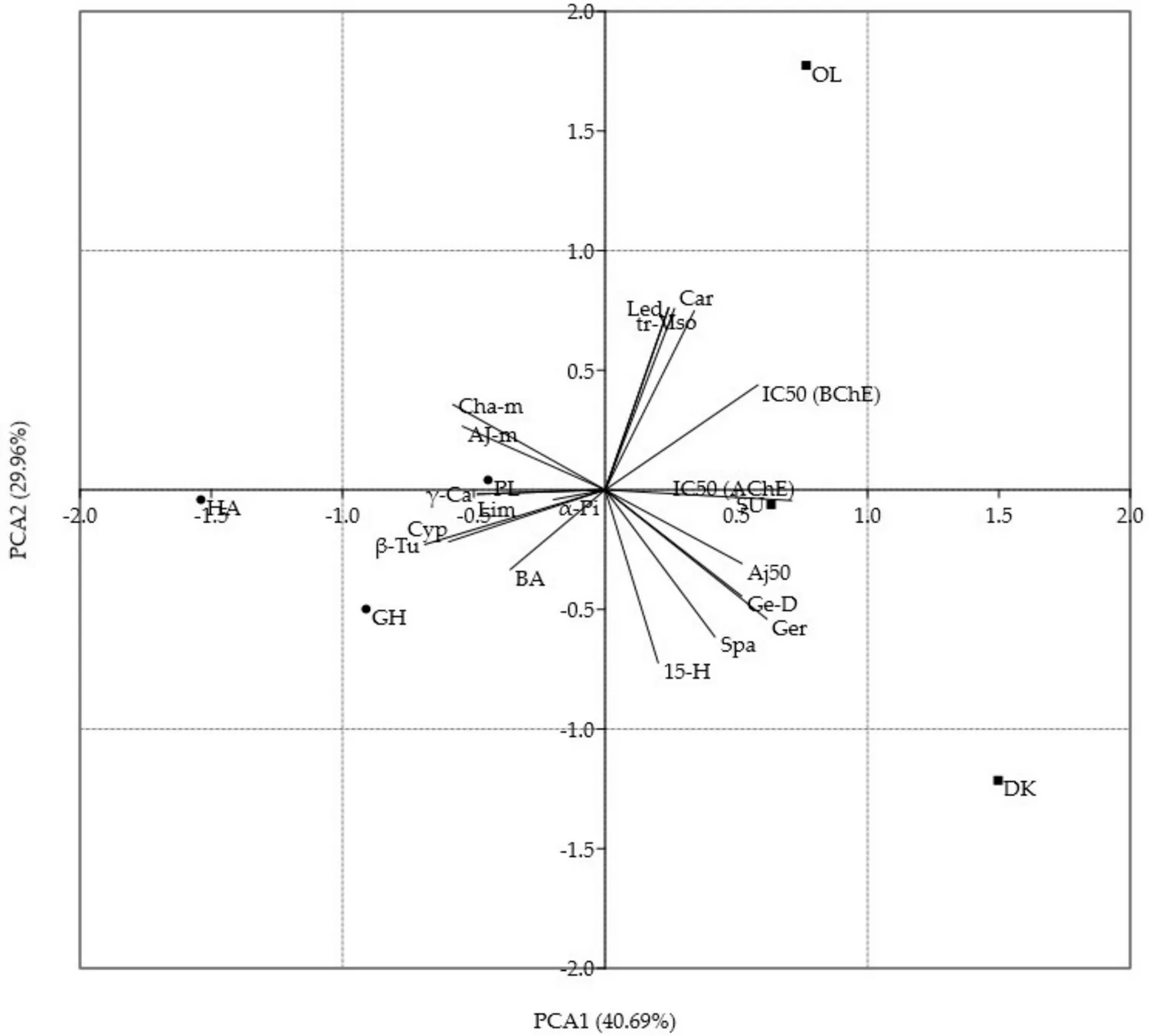
Principal Component Analysis (PCA) biplot showing the distribution of *S. canadensis* (GH, HA, PL) and *S.* × *niederederi* (DK, OL, SU) samples based on their chemical composition–representation of the dominant compounds (>5%) in the essential oils and associated response variables. Abbreviations and notes: GH, Gemerská Hôrka; HA, Hankovce; PL, Plaveč; DK, Dąbrowskie Kolonia; OL, Olecko; SU, Suwałki; Aj50/Cha50/Tt50 = LC50 of water extracts for *Ae. japonicus*/*Chaoborus spp.*/*T. tubifex*; Aj-m/Cha-m/Tt-m = mortality of *Ae. japonicus*/*Chaoborus spp.*/*T. tubifex* after application the highest essential oil dose; 15-H, 15-Hydroxy-α-muurolene; BA, Bornyl acetate; Car, Caryophyllene oxide; Cyp, Cyperenone; Ger, Germacra-4(15).5.10(14)-trien-1β-ol, Ge-D, Germacrene D; Iso, Isospathulenol; Led, Ledol; Lim, Limonene; Spa, Spathulenol; tr-V, trans-Verbenol; α-Pi, α-Pinene; β-Tu, β-Turmerone; γ-Ca, γ Cadinene; IC50 (BChE) = butyrylcholinesterase inhibition; IC50 (AChE) = acetylcholinesterase inhibition

**Fig. 4:**
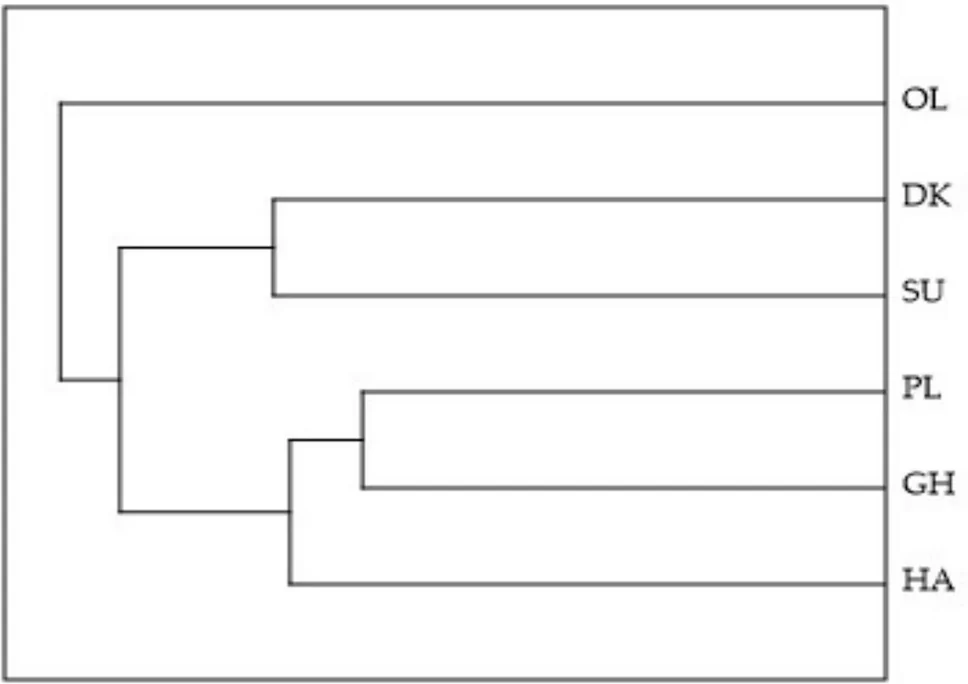
Dendrogram obtained by Hierarchical cluster analysis, Ward’s method of *S. canadensis* (GH, HA, PL) and *S.* × *niederederi* (DK, OL, SU) samples based on their chemical composition–representation of the dominant compounds (>5%) in the essential oils. Abbreviations and notes: GH, Gemerská Hôrka; HA, Hankovce; PL, Plaveč; DK, Dąbrowskie Kolonia; OL, Olecko; SU, Suwałki

Sample scores and Hierarchical Cluster Analysis, Ward’s method consistently indicated separation between goldenrod species, with *S. canadensis* localities tending to occur on the negative side of PC1 and *S.* × *niederederi* samples on the positive side (Fig. 3), forming two distinct clusters corresponding to goldenrod species (Fig. 4).

The performed analyses indicated a clear separation between *S. canadensis* and *S.* × *niederederi*, suggesting that differences in the compounds representation may contribute to the observed variation in essential oils activity.

## Conclusion

The present study demonstrated that the biological activity of *Solidago canadensis* and its hybrid *S.* × *niederederi* strongly depends on both extract type and target organism. Essential oils exhibited pronounced larvicidal activity against the invasive mosquito *Aedes japonicus*, with LC₅₀ values below 0.06 mg/mL, whereas water extracts were considerably less active. In contrast, water extracts showed high toxicity toward annelids, particularly *Eisenia fetida*, while essential oils were largely non-toxic to earthworms and *Tubifex tubifex* but exhibited unexpectedly high toxicity toward *Chaoborus* spp., highlighting marked differences in species-specific susceptibility among non-target invertebrates.

Chemical profiling combined with multivariate analyses revealed clear separation between *S. canadensis* and *S.* × *niederederi*, reflecting species-specific differences in secondary metabolite composition. Correlation analyses suggested that caffeic acid, *p*-hydroxybenzoic acid and saponins may contribute to the biological activity of water extracts, whereas bornyl acetate was associated with stronger cholinesterase inhibition in essential oils. However, these relationships should be interpreted cautiously, as the present study does not establish direct causal links between individual compounds and the observed biological effects.

Beyond their potential application as natural larvicides against *A. japonicus*, our findings suggest that invasive *Solidago* species may also represent an overlooked source of ecologically relevant bioactive compounds. The pronounced toxicity of water extracts toward selected non-target invertebrates indicates that dense *Solidago* stands could locally influence soil and aquatic invertebrate communities through the release of allelochemicals and other secondary metabolites during rainfall, litter decomposition, and seasonal biomass turnover. Although the concentrations of these compounds under natural conditions remain unknown, chemically mediated effects on non-target fauna may represent a previously underestimated mechanism by which invasive goldenrods alter ecosystem functioning and biodiversity. Future studies should therefore focus on identifying the compounds responsible for these effects, elucidating their modes of action, and evaluating their environmental fate, persistence, and ecological relevance under field conditions.

## Data Availability

The data can be obtained upon request from the readers.
